# Mapping rare, deleterious mutations in Factor H: Association with early onset, drusen burden, and lower antigenic levels in familial AMD

**DOI:** 10.1038/srep31531

**Published:** 2016-08-30

**Authors:** Erin K. Wagner, Soumya Raychaudhuri, Mercedes B. Villalonga, Anuja Java, Michael P. Triebwasser, Mark J. Daly, John P. Atkinson, Johanna M. Seddon

**Affiliations:** 1 Ophthalmic Epidemiology and Genetics Service, New England Eye Center, Tufts Medical Center, Boston, MA 02111, USA; 2 Department of Ophthalmology, Tufts University School of Medicine, Boston, MA 02111, USA; 3 Program in Medical and Population Genetics, Broad Institute, Cambridge, MA 02142, USA; 4 Partners HealthCare Center for Personalized Genetic Medicine, Boston, MA 02115, USA; 5 Division of Genetics, Brigham and Women’s Hospital, Boston, MA 02115, USA; 6 Division of Rheumatology, Immunology, and Allergy, Brigham and Women’s Hospital, Boston, MA 02115, USA; 7 Faculty of Medical and Human Sciences, University of Manchester, Manchester M13 9PL, UK; 8 Washington University School of Medicine, Department of Medicine, Division of Nephrology, Saint Louis, MO 63110, USA; 9 Washington University School of Medicine, Department of Medicine, Division of Rheumatology, Saint Louis, MO 63110, USA; 10 Analytic and Translational Genetics Unit, Massachusetts General Hospital, Boston, MA 02114, USA; 11 Sackler School of Graduate Biomedical Sciences, Tufts University, Boston, MA 02111, USA

## Abstract

The genetic architecture of age-related macular degeneration (AMD) involves numerous genetic variants, both common and rare, in the coding region of complement factor H (*CFH*). While these variants explain high disease burden in some families, they fail to explain the pathology in all. We selected families whose AMD was unexplained by known variants and performed whole exome sequencing to probe for other rare, highly penetrant variants. We identified four rare loss-of-function variants in *CFH* associated with AMD. Missense variant *CFH* 1:196646753 (C192F) segregated perfectly within a family characterized by advanced AMD and drusen temporal to the macula. Two families, each comprising a pair of affected siblings with extensive extramacular drusen, carried essential splice site variant *CFH* 1:196648924 (IVS6+1G>A) or missense variant rs139360826 (R175P). In a fourth family, missense variant rs121913058 (R127H) was associated with AMD. Most carriers had early onset bilateral advanced AMD and extramacular drusen. Carriers tended to have low serum Factor H levels, especially carriers of the splice variant. One missense variant (R127H) has been previously shown not to be secreted. The two other missense variants were produced recombinantly: compared to wild type, one (R175P) had no functional activity and the other (C192F) had decreased secretion.

Age-related macular degeneration (AMD), an irreversible degenerative disease, is the leading cause of blindness in adults over the age of 60. The disease affects the central region of the retina, resulting in progressive visual impairment and reduced quality of life. AMD is highly heritable and twin studies have shown that between 46% and 71% of phenotypic variance is explained by genetic factors^1^,^2^. Several environmental and genetic components contribute to its multifactorial etiology^2^.

Early genome-wide scans for evidence of linkage in AMD families revealed several signals including one mapping to the long (q) arm of chromosome 1^3^,^4^,^5^. Investigation of the genetic architecture of AMD subsequently evolved over the next 15 years on population, family, and individual levels. Genome-wide association studies (GWAS) and meta-GWAS efforts identified numerous genetic variants in over 20 different genes showing an association with AMD risk, including a common variant in the coding region of complement factor H (*CFH*) (RefSeq NM_000186)^6^,^7^,^8^,^9^,^10^,^11^,^12^,^13^. While these known variants may explain high burden of disease in some AMD families, they do not explain the patterns of disease observed in several other families with multiple affected individuals.

Recent studies have identified several rare, functional, penetrant variants in genes involved in the alternative pathway (AP) of complement activation that are associated with high risk of AMD and earlier age of disease onset. The first rare variant identified to be associated with AMD, *CFH* R1210C, was found to be highly penetrant with an odds ratio of about 20 (P = 7.0 × 10^−6^)^14^. Other rare variants in the *CFH* gene, including R53C, D90G, and P503A, were later found to segregate within AMD families densely affected with the disease^15^,^16^. A recent targeted sequencing study of independent cases and controls showed an enrichment of rare *CFH* variants in AMD patients^17^. Rare variants in *CFI*, *C3*, and *C9* have also been identified by targeted sequencing of the exons of these genes in a sample of over 7,600 AMD cases and unaffected controls^18^. Results from this and other recent studies showed a burden of *CFI* rare variants in AMD cases^18^,^19^, an association between AMD and the rare K155Q variant in *C3*^18^,^20^,^21^, and an association between AMD and the rare P167S variant in *C9*^18^.

Considering the increased risk of AMD associated with rare variants discovered to date, we selected densely affected families with a lower than expected genetic load for known AMD-associated SNPs using an established method^22^. We conducted whole exome sequencing to investigate the coding sequences of genes and to determine whether rare and highly penetrant variants could explain the prevalence of AMD in these families. We sought to examine the functional and phenotypic consequences of identified rare variants in order to understand their role in the onset and development of AMD.

## Results

Herein we report four independent families each harboring a unique rare, loss-of-function (LOF) variant in *CFH* associated with AMD. These variants were identified using the xBrowse software to filter variants by the following stringent criteria: (1) had a minor allele frequency (MAF) of <0.1% in the 1000 Genomes Project and Exome Aggregation Consortium (ExAC) databases, (2) belonged to a potentially damaging functional annotation class, (3) was predicted *in silico* to have a functional impact on protein function, and (4) followed the inheritance pattern of the disease within the family.

**Figure 1:**
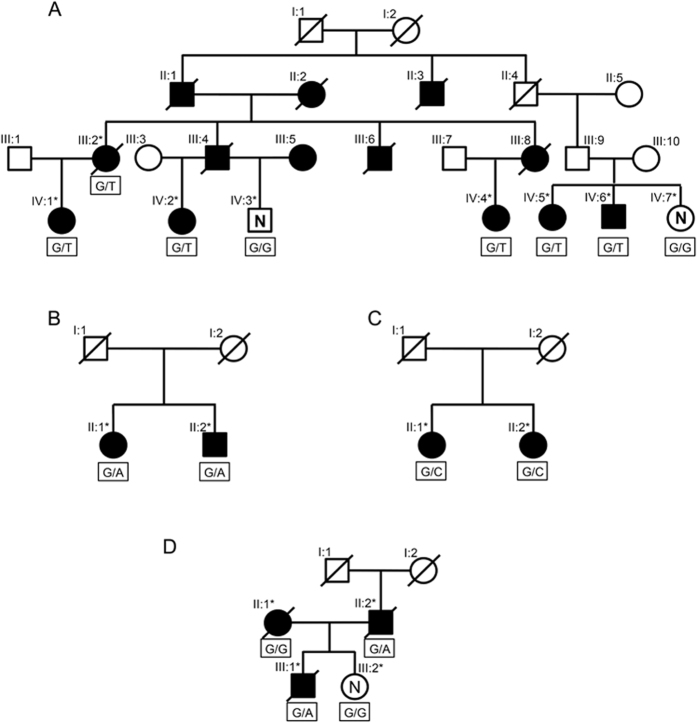
Pedigree diagrams for families carrying rare, loss-of-function *CFH* variants. (**A**) Pedigree **A** (*CFH* C192F), (**B**) Pedigree **B** (*CFH* IVS6 + 1G > A), (**C**) Pedigree **C** (*CFH* R175P), (**D**) Pedigree **D** (*CFH* R127H); ⚪ = female; ◽ = male; * = sequenced; ∅ = deceased; ⦁ and ◾ = affected with advanced AMD; “N” = confirmed unaffected; empty ⚪ and ◽  = unknown affection status; rare variant genotype listed below each sequenced subject.

Fig. 1A shows a multigenerational pedigree with both AMD-affected and unaffected members from which we sequenced eight subjects from two generations. Affected and unaffected family members were classified according to the Clinical Age-Related Maculopathy Staging (CARMS) system (see Methods)^23^. Affected family members showed early age at onset of AMD with a mean age at first diagnosis of 58 and a range between 46 and 67 years of age. The unaffected members (Pedigree A; IV:3 and IV:7) were 67 and 65 years old at their most recent follow-up exams, respectively, and were confirmed by ocular examination to be unaffected. After applying our described variant filtering criteria to Pedigree A (Table 1), only one rare non-synonymous variant segregated with the autosomal dominant pattern of inheritance suggested for AMD in this family. The variant, *CFH* 1:196646753 is a missense mutation that results in the substitution of a phenylalanine for the cysteine at position 192 of the protein (*CFH* C192F). The risk allele for this variant has a frequency of 0 in the 1000 Genomes Project and ExAC databases. PolyPhen-2, SIFT, MutationTaster2, and FATHMM all predict this mutation to be deleterious to the protein structure. We selected this family based on low genetic risk scores for members affected with AMD and we verified that no other *CFH* variants segregated with AMD within the family. Though all family members carried the more common, lower impact AMD risk alleles at *CFH* rs1061170 (Y402H) and *CFH* rs1410996, neither variant segregated with AMD in this family; in fact, all but two of the sequenced members, including both unaffected family members, were homozygous for the risk allele at *CFH* rs1410996. Thus, these known common variants did not segregate with the disease and could not explain the high level of penetrance of disease observed in this family as effectively as the rare C192F variant. Factor H (FH) bearing this variant was recombinantly produced on two separate transfections and found to be secreted in the first transfection at 36% and in the second at 57% of the amount compared to wild type (Supplementary Fig. S1).

Pedigree B (Fig. 1B) shows a pair of siblings affected with geographic atrophy. Filtering common variants and variants with low function impact on the protein left 114 variants shared by both siblings. Of those, a rare essential splice site variant in *CFH* emerged as a putative candidate mutation to explain the prevalence of AMD in this family. This mutation, *CFH* 1:196648924, is a substitution of an adenine for a guanine at the first base pair of the sixth intron of *CFH* and is located in a splice donor site (*CFH* IVS6 + 1G > A). It is predicted to be “disease causing” by MutationTaster2 and to cause an “alteration of the wild type donor site, most probably affecting splicing” by Human Splicing Finder. The risk allele for this variant has a frequency of 0 in the 1000 Genomes Project and ExAC databases. Both affected members were heterozygous for the risk allele at *CFH* Y402H and *CFH* rs1410996, although these variants are unlikely to explain the severity of disease seen in this family. The splice site variant observed in this family is located at the 5′ end of the sixth intron. Retention of this intron results in the addition of 30 amino acids to the protein before a stop codon is encountered, leaving a truncated protein 20% the size of the wild type protein with only the first four CCP domains. The truncation caused by this mutation in an essential splice site likely explains the low serum FH levels measured in the two members of this family as described below.

Pedigree C (Fig. 1C) shows a pair of siblings with advanced AMD. After filtering of common variants and variants with low potential for impacting protein function, 108 variants remained for analysis. Of these, 97 were missense variants and 4 were variants in essential splice sites. A single missense variant in *CFH* was identified as a probable candidate for the causative mutation in this family. The variant, rs139360826 (*CFH* R175P), is a substitution of a guanine for a cytosine resulting in a change of the 175^th^ amino acid from an arginine to a proline. It is predicted to be “possibly damaging” by PolyPhen2 and “damaging” by SIFT, while MutationTaster2 and FATHMM predict the variant to be neutral. This variant has a minor allele frequency of 0 in the 1000 Genomes Project and ExAC databases. Both members of this family were heterozygous for the risk allele at *CFH* Y402H and *CFH* rs1410996, although these variants are unlikely to explain the severity of disease seen in this family. Factor H bearing this rare variant was produced recombinantly and it had no cofactor activity for C3b (Supplementary Fig. S2).

Pedigree D (Fig. 1D) shows a family with two generations affected with advanced AMD. We found 34 rare, high impact variants segregating with AMD in individual II:2 and his son (III:1). Of these variants, a rare missense variant in *CFH* was identified as the candidate variant explaining AMD on the paternal side of the family. This variant, rs121913058 (*CFH* R127H), is a substitution of a guanine to adenine that results in the change from an arginine to histidine at the 127^th^ amino acid. This variant is predicted to be deleterious to the protein structure according to all four *in silico* prediction software programs. In accord with these predictions, this variant has been extensively evaluated and shown not to be secreted^24^. It has a frequency of 3.0 × 10^−5^ (an allele count of 2/66,622) in the European samples from the ExAC database and a frequency of 0 in the 1000 Genomes Project. Both subject II:1 and her unaffected daughter, subject III:2, were heterozygous for the risk allele at *CFH* Y402H and *CFH* rs1410996, thus these variants did not segregate with AMD on the maternal side of this family. Subject II:2 is heterozygous for the risk allele at *CFH* rs1410996 but homozygous for the non-risk allele at *CFH* Y402H. The affected son, subject III:1, is heterozygous for the risk allele at *CFH* Y402H and homozygous for the risk allele at *CFH* rs1410996. Considering the pattern of risk allele inheritance of the two common variants in *CFH*, there is little evidence they are responsible for the AMD seen in the affected family members.

We assessed the genotypes of all family members for known AMD and other retinal disease-associated variants to ensure that the disease seen in affected subjects could not be explained by other known high impact risk alleles. None of the family members carried the rare AMD risk alleles at *CFH* R53C, *CFH* D90G, *CFH* P503A, or *CFH* R1210C^14^,^15^,^16^,^18^ nor did they carry risk alleles for macular diseases in the *BEST1*, *ABCA4*, or other retinal degeneration-associated genes^25^.

**Figure 2:**
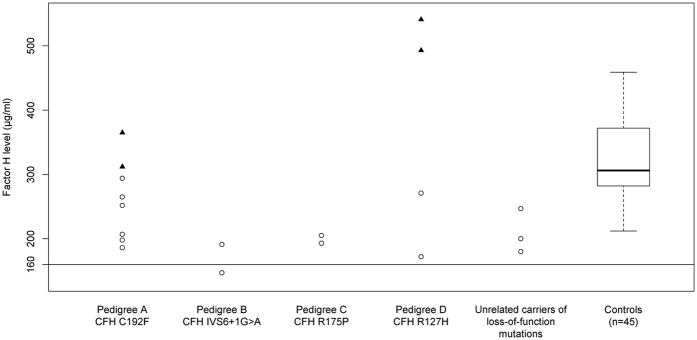
Serum factor H levels according to carrier state of rare *CFH* variants. ⚪ = subjects carrying rare *CFH* variant; ▴ = subjects not carrying rare *CFH* variant. controls = CARMS grade 1 and no known rare variants in *CFH*; unrelated carriers of loss-of-function mutations = nonsense, splice-site, and loss of a conserved cysteine; normal clinical laboratory range = 160–412 μg/ml.

We measured serum FH levels in each family in order to assess the effects of the four *CFH* variants on secretion of the FH protein. Members of all four pedigrees who carried rare, high impact *CFH* variants had lower serum antigenic FH levels compared to family members who did not carry the variants. Their levels were still within the normal clinical laboratory range (160–412 μg/ml), with the exception of one member of Pedigree B (Pedigree B; II:1 = 147 μg/ml) (Fig. 2 and Supplementary Table S1).

We selected serum samples from our biorepository to determine if these relatively low but normal levels are characteristic of individuals who are heterozygous for rare LOF *CFH* variants. We first selected serum from unrelated individuals who were heterozygous for at least one rare LOF *CFH* variant (n = 3), and additionally selected serum from a set of unaffected control individuals (CARMS grade 1 in the worse eye) who were confirmed not to carry any of the known rare variants in *CFH* (n = 45). The mean serum FH level in the unaffected controls was significantly different from mean serum FH level in the unrelated carriers (P = 0.01). Rare variant carriers in Pedigrees A, B, and C also had serum FH levels that were significantly different from unaffected controls (P = 1.0 × 10^−3^, 4.3 × 10^−2^, and 4.58 × 10^−8^, respectively). There was no significant difference in mean serum FH level between rare variant carriers from Pedigree D and unaffected controls (P = 0.26), but there were only two carriers in this family and for one, the serum FH level was lower than the controls. We further confirmed that serum FH levels of rare variant carriers from all four families did not differ from the serum FH levels for unrelated LOF variant heterozygotes (P values: 0.29 to 0.84) (Fig. 2).

We used our AMD genotype-phenotype database to explore the clinical history and phenotypic appearance of carriers and non-carriers of each rare variant. In addition to these variants being associated with more advanced AMD within these families, carriers also had earlier age of onset of advanced disease (mean age = 59.2) compared to individuals with advanced AMD without rare *CFH* variants (mean age at diagnosis in our AMD database = 69.6, n = 1,627)^26^. We also explored disease symmetry, and among affected individuals carrying a rare *CFH* variant in these families, 75% (n = 9 of 12 carriers) exhibited a symmetric fundus phenotype.

**Figure 3:**
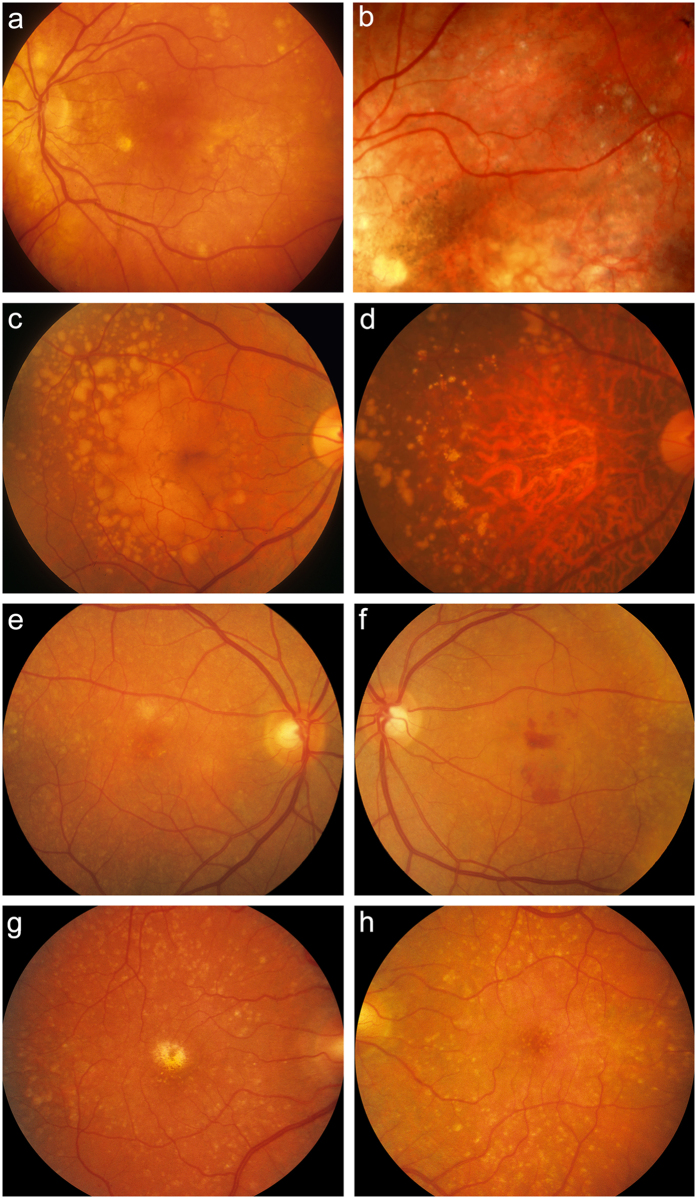
Fundus photographs from family members carrying rare *CFH* variants showing numerous large drusen and extramacular drusen. Fundus color photographs of subjects in the four pedigrees: IV:5 from Pedigree A showing several large macular and extramacular drusen with retinal pigment epithelial irregularities in her left eye (OS) at age 71 (**a**) and extramacular drusen and macular pigment disruption following intravitreal anti-vascular endothelial growth factor injections OS after 8 years of follow-up (**b**). II:1 from Pedigree B showing numerous large macular and extramacular drusen with transition in her right eye (OD) from drusenoid retinal pigment epithelial detachments at age 64 (**c**) to geographic atrophy at age 76 (**d**). Subject II:2 from Pedigree C showing several drusen throughout the posterior pole OD (**e**) and OS (**f**) at age 55, with neovascular disease OS. Subject III:1 from Pedigree D showing numerous drusen OD at age 56 (**g**) and OS at age 54 (**h**); patient previously received laser treatment OD for neovascular macular degeneration. Some subjects progressed after date of images; last known phenotypes are shown in Table 2.

Segregation with extramacular drusen was seen in both eyes of all affected family members in Pedigrees A-C (Table 2 and Fig. 3). All members of Pedigree A carrying the *CFH* C192F variant showed several large macular and extramacular drusen in the region temporal to the macula. Members of Pedigree B carrying the *CFH* IVS6 + 1G>A variant had extensive extramacular drusen in four of the seven possible peripheral fundus locations. Both members of Pedigree C carrying *CFH* R175P had extensive extramacular drusen in all seven peripheral fundus locations. We investigated the presence of one or more locations of extramacular drusen versus none by comparing the subjects in these three families who had extramacular drusen to 230 unrelated individuals with advanced disease and complete extramacular drusen information in our database who did not carry the *CFH* C192F, *CFH* IVS6+1G>A, or *CFH* R175P variants based on sequencing. Overall, 57% of the unrelated individuals without these variants had extramacular drusen, whereas 100% of individuals within the three families who carried these variants exhibited drusen outside the macular area (P = 6.1 × 10^−3^). Extramacular drusen burden did not appear to segregate with the presence of the rare variant *CFH* R127H seen in Pedigree D (Table 2). Subject II:2 of Pedigree D, a carrier of the risk allele, had no drusen in any of the seven peripheral fundus locations based on available imaging, while his son, also a carrier of the risk allele, had extensive extramacular drusen in all seven locations.

## Discussion

Using next-generation whole exome sequencing and a step-wise filtering methodology, we identified highly penetrant *CFH* variants that were strongly associated with advanced AMD in four independent families with a low burden of risk based on previously reported AMD genetic variants^22^. The identified variants were associated with a higher frequency of drusen, earlier age of disease onset, and phenotypic symmetry compared with non-carriers. Regarding the four mutant proteins, expression profiles and functional analyses provided an explanation for low serum FH levels and/or deficient cofactor activity.

The FH protein consists entirely of 20 homologous repeating units (“like beads on a string”) of from 56 to 66 amino acids each. They are variably known as a complement control protein (CCP), short consensus repeats (SCR), or Sushi domains. Most are encoded by a single exon. Each CCP harbors four cysteine residues that participate in two disulphide bridges with one at each end of the repeat. Repeats with a missense mutation involving one of the four cysteine residues commonly lead to a misfolded domain, and the protein is poorly secreted or has a short half-life leading to haploinsufficiency. The variant C192F (Pedigree A) is of the aforementioned category. We prepared this variant recombinantly in the laboratory and found that the protein was secreted at ~45% the amount compared to wild type. We also prepared the variant R175P (Pedigree C) recombinantly and found that it had no functional activity. Most splice site variants, like IVS6 + 1G>A in Pedigree B, lead to haploinsufficiency as the protein is not synthesized. This explains the low antigenic levels observed in this family. The fourth variant, R127H (Pedigree D), has been reported in the literature and shown not to be secreted. We believe carriers in these families have only one allele substantially contributing to the blood level of FH. The protein from the other allele is either not synthesized/secreted or secreted but having moderately reduced function. Thus, at sites of injury and/or degeneration, complement homeostasis is altered such that overactivation of the AP occurs and thereby generates excessive and potentially damaging anaphylatoxins (C3a and C5a) and membrane attack complexes (C5b-C9) in the retina.

Variants in *CFH* associated with AMD, including two common variants rs1061170 and rs1410996, were initially identified using case-control study designs^8^,^13^,^27^. These two common variants explain 17% of AMD liability, but not everyone affected with AMD carries these variants^14^. Rare variants in *CFH* as well as other genes in the complement pathway have since been identified, and carry a higher risk of disease^14^,^15^,^16^,^17^,^18^. The *CFH* R53C variant, located in CCP1, decreases the ability of FH to perform decay accelerating activity^15^; the *CFH* D90G variant, located in CCP2, was found to decrease cofactor-mediated inactivation^15^; and the *CFH* R1210C variant, located in CCP20, shows defective binding of FH to C3d, C3b, heparin/glycosaminoglycans, and endothelial cells (Table 3)^14^,^30^. Another rare variant was found to segregate with AMD in an Amish family, but no functional work was done to determine the effect of the mutation on FH protein. It is located in a CCP whose function is currently unknown^16^. Other variants in *CFH* were associated with basal laminar drusen^34^ but no functional work was done to assess the effect of these variants. In a separate targeted sequencing study of unrelated cases and controls, we found 65 rare variants in *CFH*^17^. The four variants found to segregate with AMD in this family-based study were identified independent of that study and with a different next generation whole exome methodology. These four variants were discovered to be a subset of the variants found with targeted sequencing of cases and controls. Thus, the rare variants were confirmed using two different platforms. The association in these families, the phenotypic appearance of the carriers, and the functional impact of the variants underscore their importance.

Previous studies report a distinctive drusen appearance as well as an earlier age of AMD onset in carriers of rare variants in *CFH*; our results are consistent with these findings^15^,^25^. Carriers of the four rare *CFH* variants we report here showed an earlier age of disease onset when compared to other cases in our AMD registry. Extramacular drusen, seen in 11 of 12 carriers of variants in this study, is not a clinical phenotype consistently seen in AMD, but it has been reported as associated with other mutations in *CFH*^25^.

Of note, 9 of the 12 carriers of a rare *CFH* variant in these families had similar phenotypic appearances in both eyes, a characteristic trait of monogenic ocular diseases and macular dystrophies. These mutations and their associated phenotypic characteristics may be helpful in identifying individuals with macular degeneration in a clinical population who are more likely to be carriers of these rare variants.

Access to a large family-based resource with clinical phenotype data and family history information gave us the ability to identify variants that are rare in the general population but enriched in certain families. We chose to utilize whole exome sequencing for a subset of our vast database in order to examine coding regions across the genome. This approach also allowed us to confidently exclude other candidate variants if they did not meet our strict filtering criteria implemented through the xBrowse software. Serum protein antigenic and functional analyses reflected the structural impact of these rare LOF mutations. These data strengthen our conclusion that these mutations are responsible for the AMD phenotype characterized in these families.

Identification of these rare variants augments our understanding of the biology of FH which could potentiate the development and optimization of novel treatments aimed at slowing the progression of AMD and decreasing visual loss. These mutations often result in haploinsufficient protein levels, and selected patients may respond to treatments that involve FH supplementation. Earlier and more frequent monitoring for the initial signs of AMD in young members of families carrying these variants could lead to better management and education regarding behavioral risk factors and ultimately a reduction in the ocular morbidity associated with the disease. Risk prediction models of AMD progression have shown that, in addition to eight common AMD risk variants, the rare variants *CFH* R1210C and *C3* K155Q are also independently associated with progression of the disease to advanced stages^36^. Incorporation of additional rare or low frequency variants may aid in the development of a more precise model to better predict a patient’s risk of developing advanced stages of AMD. Furthermore, growing evidence of the impact of rare variants may raise awareness in the clinic setting for high risk of disease in the families affected. Investigating the associated functional and phenotypic consequences of rare variants will further our understanding of their role and that of *CFH* in the pathophysiology of AMD, and may lead to innovative therapeutic techniques.

## Materials and Methods

### Study population

All study participants were previously enrolled in ongoing genetic and epidemiologic studies of AMD. Approval for this research was obtained from the institutional review board at Tufts Medical Center. Signed informed consent was obtained from all participants, and all procedures were carried out in accordance with approved protocols.

### Diagnosis and phenotyping

All affected and unaffected individuals in the study were evaluated by board-certified ophthalmologists. Individuals either (1) were clinically evaluated with visual acuity measurements, dilated slit lamp biomicroscopy, and stereoscopic color fundus photography or (2) had ophthalmologic medical records and images reviewed by retina specialists. Affected individuals had clinical evidence of AMD classified as drusenoid retinal pigment epithelial detachment, geographic atrophy (advanced dry AMD), or neovascular AMD (wet AMD) according to CARMS grades 3B, 4, and 5, respectively^23^. We defined unaffected individuals as those who had no signs of early, intermediate, or advanced macular degeneration in either eye and were categorized as CARMS grade 1 in both eyes. We reviewed ocular records, fundus photographs, and other ocular images including autofluorescence and ocular coherence tomography to determine the grade.

We evaluated the presence of drusen in the macular area (defined as a circular area of 3 mm or 2 disc diameters in radius, centered at the fovea) from color fundus images which were obtained in up to seven standard fields based on the modified Airlie House classification^38^. With standard field 2 centered on the macula, the presence of drusen in the macular area was assessed in this field as previously described^25^. We also reviewed fundus images to evaluate the presence of drusen in seven extramacular regions of the retina including temporal to the macula, nasal to the optic disc (defined as a semicircular area of 3 mm or 2 disc diameters in radius, nasal to the optic disc), along the temporal vascular arcade, and the superotemporal, inferotemporal, superonasal, and inferonasal fundus quadrants (defined as the retina beyond the retinal vascular arcades extending to or beyond the equator). We evaluated each region for the presence of drusen as previously described^25^.

### Family selection

We selected families whose burden of AMD could not be explained by any of the known AMD-associated common or rare variants^22^. We selected individuals based on the following criteria: (1) families had multiple members affected with macular degeneration; (2) multiple affected individuals with a low genetic risk score and relatively younger age of onset of AMD (less than 75); and (3) those affected did not carry risk alleles at *CFH* R1210C, *CFH* R53G, *CFH* D90G, *CFH* P503A, and *C3* K155Q. The genetic risk score is defined as the sum of the log odds ratio for each risk allele from 26 loci associated with AMD (equation (1)):


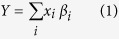


where x_i_ is 0, 1, or 2 copies of the 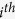 risk alleles and 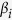 is the log of the odds ratio of the 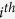 risk allele estimated using multivariate logistic regression on genetic data collected on a cohort of over 4,800 independent AMD cases and validated controls. A low genetic risk score is defined as any score that is lower than the risk score represented by the maximized sum of sensitivity and specificity (threshold score = 0.874) (Supplementary Fig. S3).

### Exome sequencing

Genomic DNA was extracted from blood using the standard protocol for purification of DNA from up to 10 ml of whole blood samples using the Qiagen Autopure LS automated Nucleic Acid Purification Instrument. Exome sequencing was performed at the Perkin Elmer Center for Genome Innovation at University of Connecticut. Genomic DNA was prepared and exomic sequence was targeted following the SureSelectXT Target Enrichment System for Illumina Paired-End Sequencing Library 6.1 protocol from Agilent. Following exome library preparation, the samples were sequenced using the Illumina HiSeq2000 Sequencing System. The sequenced samples had an average of 96.6% of the exome covered at ≥10X. After quality control and variant calling, 598,065 high quality variants were identified.

### Read mapping, variant detection, and annotation

Following deconvolution of barcodes from each lane, individual reads were aligned to the human reference genome (hg19) using the Burrows Wheeler Aligner resulting in BAM files^39^. Variant calling was performed using the best practices recommendations of the tools in the Genome Analysis Toolkit (GATK) version 3.1 suite (http://www.broadinstitute.org/gatk/guide/best-practices)^40^,^41^. Genomic variant call format (gVCF) files containing variant calls for all loci were created separately for each sample using the HaplotypeCaller tool. The gVCFs were combined and raw genotypes were called for the sample set as a whole using the GenotypeGVCFs tool. The resulting raw genotype calls in the VCF file were filtered for low quality genotypes using the Variant Quality Score Recalibration tool. Functional effects for the variants were annotated using the Ensembl Variant Effect Predictor^42^.

### Data analysis

For densely affected families with a low burden of known AMD-associated variants, we hypothesized that some of these families might be explained by variants that are highly penetrant and follow an autosomal dominant mode of inheritance. In order to test this hypothesis, we designed the following filtering strategy to identify rare and potentially pathogenic exomic variants that segregate with AMD in families. To screen for rare variants that segregate with AMD in the families, the xBrowse (https://xbrowse.broadinstitute.org) tool was used to filter variants in a step-wise manner. We prioritized variants that (1) were rare (MAF <0.1% in the 1000 Genomes Project and the ExAC databases), (2) belonged to a potentially damaging functional annotation class (i.e., nonsense, missense, splice site, frameshift), (3) were predicted to have a functional impact on protein function (i.e., *in silico* prediction of damaging or deleterious by PolyPhen-2^43^, SIFT^44^, MutationTaster2^45^, FATHMM^46^, and Human Splicing Finder^47^ depending on the type of mutation), and (4) matched the inheritance pattern of AMD within the family (e.g., if neither parent manifested the disease [based on an ocular examination], but offspring had the macular disease, then the pattern of inheritance was determined likely to be recessive rather than dominant).

Comparisons of mean serum FH levels in carriers and non-carriers were performed using an independent samples t-test. The association of extramacular drusen and variant status was determined using Fisher’s exact test; related non-carriers from Pedigrees A and D were excluded from this analysis.

### Factor H serum level quantification

Fasting blood samples were collected for all members of each family. The blood was centrifuged and serum was separated within 60 minutes of collection. The samples were frozen and stored in liquid nitrogen until testing was performed. FH serum antigenic levels were analyzed at the National Jewish Center for Immunology and Respiratory Medicine, Diagnostic Immunology and Complement Laboratory by radial immunodiffusion with anti-human antibodies (Abs) specific for FH as previously described^48^.

### Recombinant synthesis and functional analysis of Factor H variants

#### Protein expression

Point mutations were introduced in the wild type FH cDNA in the pSV vector using site-directed mutagenesis (QuikChange; Agilent Technologies) to prepare the variants R175P and C192F, and the resulting constructs were transiently transfected in 293T cells as previously described^49^,^50^. Three days after transfection, supernatants were concentrated. Quantitation and characterization of FH were performed by sandwich enzyme-linked immunosorbent assay and Western blotting.

#### Quantitation of FH secreted into the supernatant

Briefly, the capture anti-FH antibody, A254 (Quidel, USA), was coated at 1 mg/ml overnight at 4 °C and then blocked overnight at 4 °C. Dilutions of concentrated wild-type and variant FH samples and purified human FH (Complement Technologies, Inc., USA) were incubated for 1 h and then washed with PBS containing 0.05% Tween 20. Next, goat anti-human FH Ab (Quidel, USA) was applied for 1 h at 37 °C. After washing, HRP-coupled donkey anti-goat immunoglobin G (IgG, Jackson Immunoresearch, USA) was added and incubated for 1 h at 37 °C. After washing, TMB substrate (Pierce, Rockford, IL) was added and absorbance at 630 nm was assessed in an ELISA reader.

#### Cofactor assays

FH preparations (200 ng; wild type, R175P or C192F) were incubated for 30 min at 37 °C with C3b (10 ng) and Factor I (20 ng) in 15 μl of buffer (10 mM Tris, pH 7.4, 150 mM NaCl). To stop the reaction, 7 μl of 3X reducing Laemmli sample buffer was added. Samples were boiled at 95 °C for 5 min, electrophoresed on 10% Tris-glycine polyacrylaminde gels, transferred to nitrocellulose, and blocked overnight with 5% nonfat dry milk in phosphate-buffered saline. Blots were probed with a 1:5,000 dilution of goat anti-human C3 (Complement Technologies) followed by horseradish peroxidase-conjugated rabbit anti-goat IgG and developed with SuperSignal substrate (Thermo Scientific).

## Additional Information

**How to cite this article**: Wagner, E. K. *et al*. Mapping rare, deleterious mutations in Factor H: Association with early onset, drusen burden, and lower antigenic levels in familial AMD. *Sci. Rep*. **6**, 31531; doi: 10.1038/srep31531 (2016).

## Supplementary Material

Supplementary Information

## Figures and Tables

**Table 1:** Step-wise filtering of variants according to frequency in population databases, segregation pattern, functional annotation, and predicted deleteriousness.

	Pedigree A	Pedigree B	Pedigree C	Pedigree D
Affected:Unaffected ratio per family	6:2	2:0	2:0	3:1
MAF <0.1% in databases	2605	1841	1646	1845
Shared among affected but not unaffected	11	867	847	124
High and moderate impact SNPs	5	114	108	34
Missense and nonsense variants:				
Total	5	95	97	32
Probably damaging (Polyphen-2)	1	24	20	12
Deleterious (SIFT)	3	49	45*	15
Disease Causing (MutationTaster2)	3	65	54	23
Damaging (FATHMM)	1	17	18	7
Deleterious according to all four prediction softwares	1*	7	5	3*
Deleterious according to at least 3/4 prediction softwares	1	24	20	9
Essential Splice Site Variants:				
Total	0	3	4	1
Disease Causing (MutationTaster2)	NA	2	0	1
Affects Splicing (Human Splicing Finder)	NA	1	2	1
Deleterious according to both prediction softwares	NA	1*	0	1

^*^Indicates the damaging predictions for the *CFH* rare variant in the respective family.

**Table 2:** Genotype-phenotype characteristics of families carrying rare *CFH* variants.

ID	AMD Disease Status	Genotype	CARMS Grade OD	CARMS Grade OS	Drusen Location
Pedigree A (*CFH* C192F)					
III:2	Affected	G/T	4	4	Macula, temporal to the macula
IV:1	Affected	G/T	3B	3B	Macula, temporal to the macula, nasal to the optic disc, along the vascular arcade
IV:2	Affected	G/T	5B	3A	Macula, temporal to the macula
IV:3	Unaffected	G/G	1	1	None
IV:4	Affected	G/T	3B	3B	Macula, temporal to the macula
IV:5	Affected	G/T	3A	5B	Macula, temporal to the macula, along the temporal vascular arcade, superotemporal, inferotemporal, superonasal
IV:6	Affected	G/T	5B	5B	Macula, temporal to the macula
IV:7	Unaffected	G/G	1	1	None
Pedigree B (*CFH* IVS6+1G>A)					
II:1	Affected	G/A	4	4	Macula, temporal to the macula, nasal to the optic disc, along the temporal vascular arcade, superotemporal, superonasal, inferonasal
II:2	Affected	G/A	4	4	Macula, temporal to the macula, nasal to the optic disc, along the temporal vascular arcade, superotemporal
Pedigree C (*CFH* R175P)					
II:1	Affected	G/C	5B	3A	Macula, temporal to the macula, nasal to the optic disc, along the temporal vascular arcade, superotemporal, inferotemporal, superonasal, inferonasal
II:2	Affected	G/C	5B	5B	Macula, temporal to the macula, nasal to the optic disc, along the temporal vascular arcade, superotemporal, inferotemporal, superonasal, inferonasal
Pedigree D (*CFH* R127H)					
II:1	Affected	G/G	5B	5B	Macula, temporal to the macula, nasal to the optic disc, along the temporal vascular arcade, superotemporal, inferotemporal, superonasal
II:2	Affected	G/A	4	4	None
III:1	Affected	G/A	5B	5B	Macula, temporal to the macula, nasal to the optic disc, along the temporal vascular arcade, superotemporal, inferotemporal, superonasal, inferonasal
III:2	Unaffected	G/G	1	1	None

AMD: Age-related macular degeneration; CARMS: Clinical Age-Related Maculopathy Staging^23^; OD: right eye; OS: left eye.

**Table 3:** Rare variants in *CFH* reported to be associated with age-related macular degeneration in families.

hg19 Position	SNP ID	Amino Acid Consequence	CCP	Function
1:196642206	NA	R53C	1	Normal FH levels; Decreases the ability of FH to perform decay accelerating activity^16^
1:196643011	NA	D90G	2	Normal serum FH levels; Decreases cofactor-mediated inactivation^16^
1:196645148	rs121913058	R127H	2	Low serum FH levels; Haploinsufficiency of serum FH
1:196646702	rs139360826	R175P	3	Low serum FH levels; Haploinsufficiency of serum FH
1:196646753	NA	C192F	3	Low serum FH levels; Haploinsufficiency of serum FH
1:196648924	NA	NA (Splice variant)	4	Low serum FH levels; Truncation of protein; Low levels of FH secreted from cell and haploinsufficiency of serum FH
1:196683035	rs570523689	P503A	8	May affect C3b binding affinity^17^
1:196716375	rs121913059	R1210C	20	Defective binding to C3d, C3b, heparin/glycosaminoglycans, and endothelial cells^26^,^27^,^28^,^29^
