# Supplementary material for: Mapping rare, deleterious mutations in Factor H: Association with early onset, drusen burden, and lower antigenic levels in familial AMD
Source: Sci Rep. 2016 Aug 30;6:31531. doi: 10.1038/srep31531 (PMC5004131; doi:10.1038/srep31531)
Supplement: Supplementary Information [file srep31531-s1.pdf]

Supplementary Information: Mapping rare, deleterious mutations in Factor H: Association with early onset, drusen burden, and lower antigenic levels in familial AMD

Erin K. Wagner[1,2]

Soumya Raychaudhuri[3,4,5,6,7]

Mercedes B. Villalonga[1]

Anuja Java[8]

Michael P. Triebwasser[9]

Mark J. Daly[3,4,10]

John P. Atkinson[9]

Johanna M. Seddon[1, 2, 11,*]

[1] Ophthalmic Epidemiology and Genetics Service, New England Eye Center, Tufts Medical Center, Boston, MA 02111, USA

[2] Department of Ophthalmology, Tufts University School of Medicine, Boston, MA 02111, USA

[3] Program in Medical and Population Genetics, Broad Institute, Cambridge, MA 02142, USA

[4] Partners HealthCare Center for Personalized Genetic Medicine, Boston, MA 02115, USA

[5] Division of Genetics, Brigham and Women's Hospital, Boston, MA 02115, USA

[6] Division of Rheumatology, Immunology, and Allergy, Brigham and Women's Hospital, Boston, MA 02115, USA

[7] Faculty of Medical and Human Sciences, University of Manchester, Manchester M13 9PL, UK

[8] Washington University School of Medicine, Department of Medicine, Division of Nephrology, Saint Louis, MO 63110, USA

[9] Washington University School of Medicine, Department of Medicine, Division of Rheumatology, Saint Louis, MO 63110, USA

[10] Analytic and Translational Genetics Unit, Massachusetts General Hospital, Boston, MA 02114, USA

[11] Sackler School of Graduate Biomedical Sciences, Tufts University, Boston, MA 02111, USA

[*] Corresponding author: Johanna M. Seddon, Tufts Medical Center, 800 Washington Street, #450, Boston, MA 02111; Tel: +1 6176369000; Fax: +1 6176365844; Email: jseddon@tuftsmedicalcenter.org

Supplementary Table S1: Factor H levels

ID	AMD Disease Status	Genotype	Factor H Levels (μg/ml)
**Pedigree A (*CFH* C192F)**			
**III:2**	Affected	G/T	198[a]
**IV:1**	Affected	G/T	265
**IV:2**	Affected	G/T	294
**IV:3**	Unaffected	G/G	365
**IV:4**	Affected	G/T	252
**IV:5**	Affected	G/T	186[a]
**IV:6**	Affected	G/T	207[a]
**IV:7**	Unaffected	G/G	312
**Pedigree B (*CFH* IVS6+1G>A)**			
**II:1**	Affected	G/A	147[a]
**II:2**	Affected	G/A	191[a]
**Pedigree C (*CFH* R175P)**			
**II:1**	Affected	G/C	205[a]
**II:2**	Affected	G/C	193[a]
**Pedigree D (*CFH* R127H)**			
**II:1**	Affected	G/G	541
**II:2**	Affected	G/A	172[a]
**III:1**	Affected	G/A	271
**III:2**	Unaffected	G/G	493

[a]Below range of serum Factor H levels measured in controls; minimum level measured in controls = 212 μg/ml. AMD: Age-related macular degeneration.

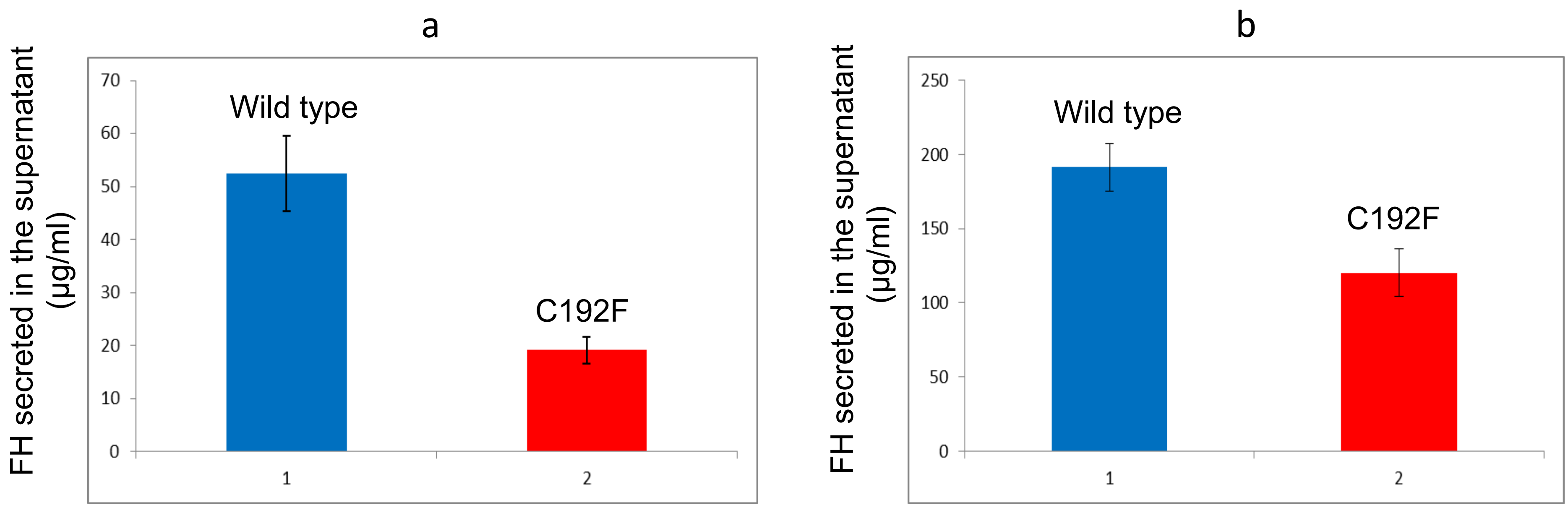

Supplementary Figure S1: Quantification of Factor H secreted into the supernatant

The wild type and Factor H (FH) variant (C192F) were recombinantly expressed in 293T cells employing two separate transfections. The quantity of wild type and variant protein was determined by ELISA.

(a) In the first transfection as shown, the mean concentration (shown as mean ± standard deviation) of wild type FH secreted into the supernatant was 52 µg/ml (± 7.1) and the mean concentration of FH C192F variant secreted was 19 µg/ml (± 2.6); i.e. the FH variant was secreted at 36% compared to wild type.

(b) In the second transfection as shown, the mean concentration of wild type FH secreted was 198 µg/ml (± 29.8) and the mean concentration of FH C192F variant secreted was 114 µg/ml (± 15.9); i.e. the FH variant was secreted at 57% compared to wild type.

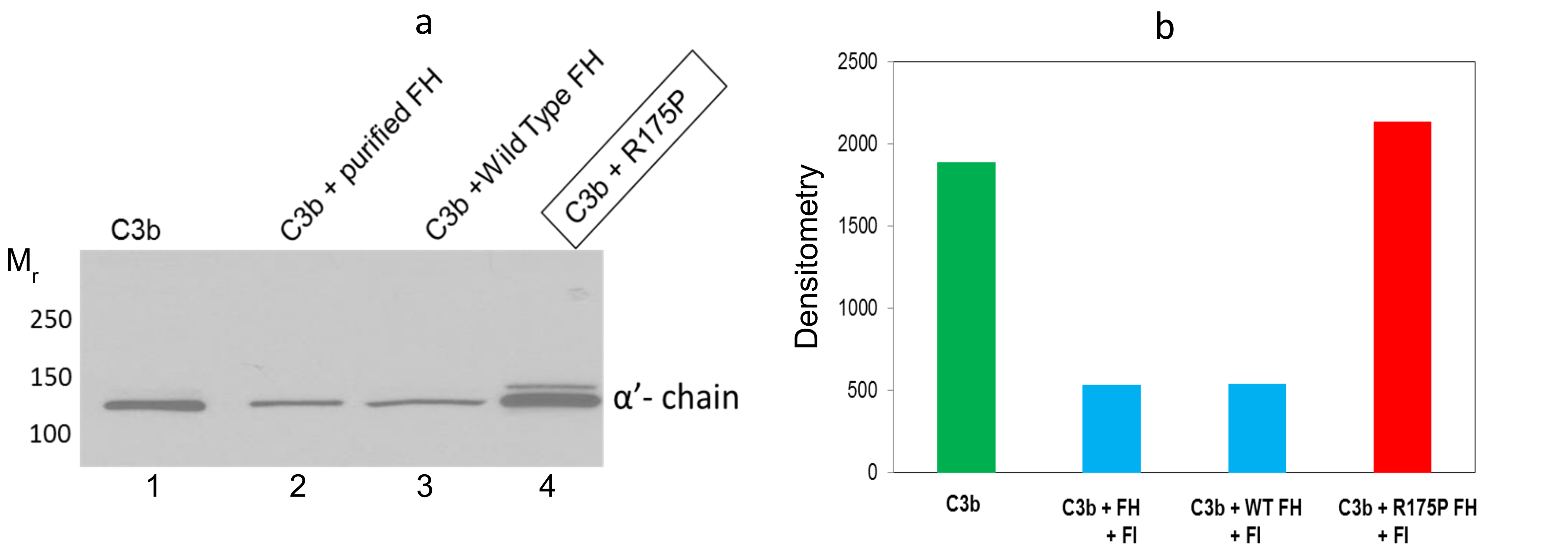

Supplementary Figure S2: Functional evaluation of a Factor H mutation: Cofactor activity

C3b is resistant to inactivation by Factor H (FH) variant R175P. We incubated the FH proteins (wild type or R175P variant) at physiological salt concentration with C3b and Factor I (FI) at 37°C for 30 min. The reaction was stopped by addition of 3x reducing buffer. After electrophoresis and transfer to a nitrocellulose membrane, Western blots were developed using goat anti-human C3 polyclonal antibody (a). Bands were quantified using densitometry (b). There was minimal, if any, detectable cleavage of C3b by FH R175P while the two positive controls (lanes 2 and 3) cleaved ~70% of the α'-chain of C3b.

Lane 1, C3b alone (10 ng, negative control); lane 2, C3b (10 ng) with purified FH (200 ng) and FI (20 ng); lane 3, C3b (10 ng) incubated with wild type FH (200 ng, recombinantly produced in our lab) and FI (20 ng); lane 4, C3b (10 ng) incubated with R175P FH variant (200 ng, recombinantly produced in our lab) and FI (20 ng). Purified C3b, FH, and FI obtained from Complement Technologies, Inc. M_r: relative electrophoretic mobility.

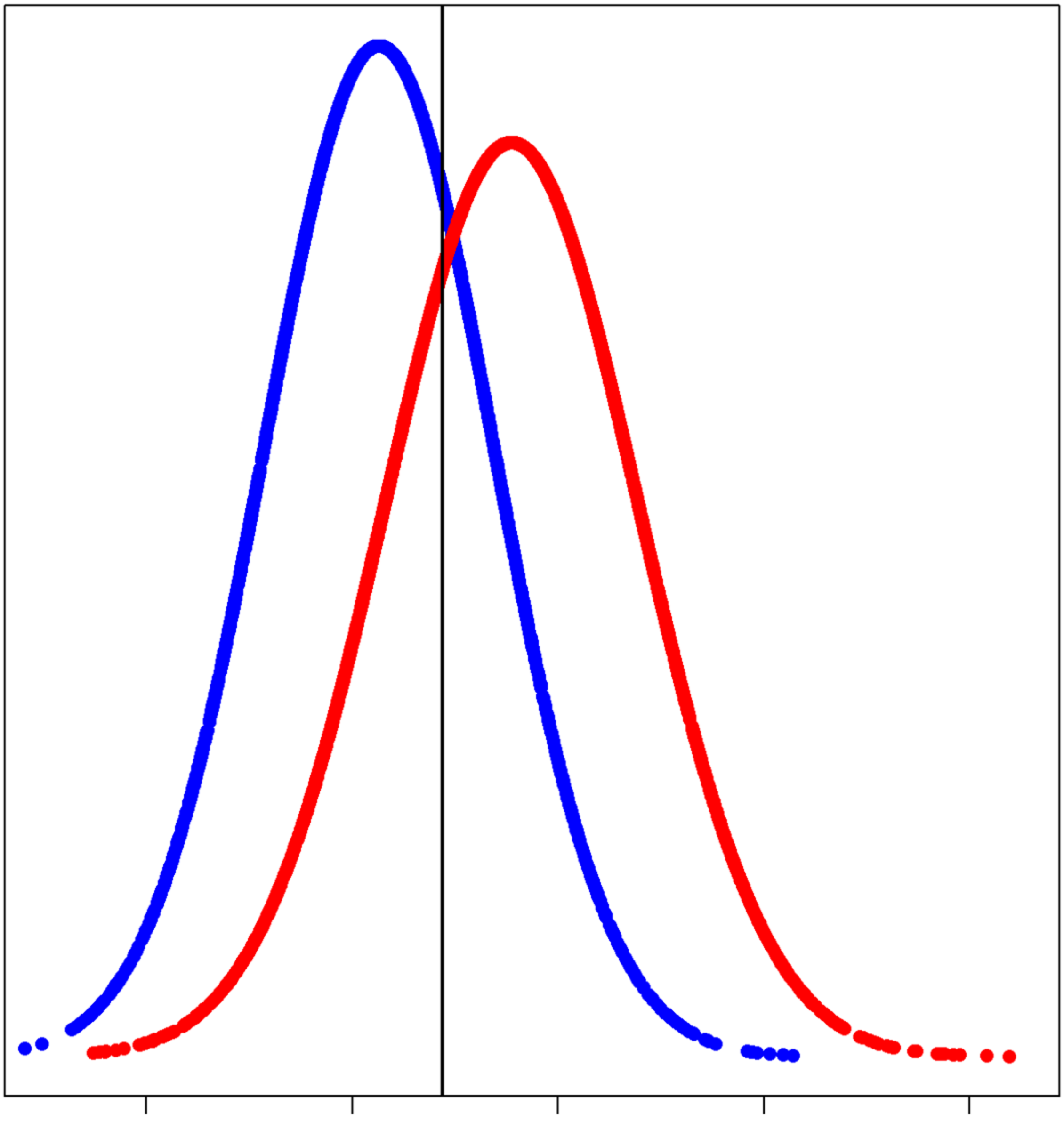

Supplementary Figure S3: Distribution of genetic risk scores

● = controls (Clinical Age-Related Maculopathy Staging [CARMS][23] grade 1); ● = AMD cases (CARMS grades 3B, 4, and 5); vertical black line indicates maximized sum of sensitivity and specificity threshold = 0.874.
